# Linaclotide induces secretion of cGMP from mouse colonic epithelium

**DOI:** 10.1186/2050-6511-14-S1-P70

**Published:** 2013-08-29

**Authors:** Boris Tchernychev, Sarah Jacobson, Caroline Kurtz, Mark Currie, Inmaculada Silos-Santiago

**Affiliations:** 1 Ironwood Pharmaceuticals Inc., Cambridge, MA 02142, USA

## 

Linaclotide is a novel receptor guanylyl cyclase C (GC-C) agonist approved for treatment of abdominal pain and constipation in patients with irritable bowel syndrome with constipation (IBS-C). Linaclotide effects on bowel movements are mediated by the intracellular cGMP that is produced upon activation of GC-C. It is hypothesized that the effects of linaclotide on abdominal pain are mediated by extracellular cGMP, which was shown to decrease the activity of pain-sensing nerves [1]. Here we used an *ex vivo* Ussing chamber assay to measure the secretion of cGMP from the mouse colonic mucosa in response to linaclotide treatment. Ion transport and epithelial barrier function were monitored by measuring short-circuit current (I_sc_) and trans-epithelial electrical resistance (TEER). Stimulation with linaclotide (1 µM) elicited a robust short-circuit current across mouse colonic epithelium. I_sc_ reached a maximum within ten minutes following stimulation with linaclotide and remained steady during the duration of the study (60 min). Treatment of colonic mucosa with linaclotide induced release of cGMP from the apical, as well as, the basolateral side of the epithelium. The time course of cGMP accumulation in the basolateral bath of the Ussing chamber was linear with an estimated cGMP secretion rate equal to 23 fmol/min×cm^2^. The trans-epithelial electrical resistance of the colonic mucosa remained high over the course of the study indicating that the barrier to diffusion of cGMP between apical and basolateral sides remained intact throughout the study.. In summary, these data demonstrate that linaclotide-stimulated mouse colonic epithelium secretes cGMP from both the apical and basolateral sides and that cGMP is available in the submucosal interstitial space to inhibit colonic nociceptors.
